# Carbapenemase Production and Epidemiological Characteristics of Carbapenem-Resistant *Klebsiella pneumoniae* in Western Chongqing, China

**DOI:** 10.3389/fcimb.2021.775740

**Published:** 2022-01-04

**Authors:** Wan Huang, Jisheng Zhang, Lingyi Zeng, Chengru Yang, Lining Yin, Jianmin Wang, Jie Li, Xinhui Li, Kewang Hu, Xiaoli Zhang, Beizhong Liu

**Affiliations:** ^1^ Department of Microbiology, Yongchuan Hospital of Chongqing Medical University, Chongqing, China; ^2^ Department of Microbiology, Jiaxing Maternity and Child Health Care Hospital, Jiaxing, China; ^3^ Department of Microbiology, The First Affiliated Hospital of Jiamusi University, Jiamusi, China; ^4^ Department of Microbiology, Affiliated Hangzhou Xixi Hospital, Zhejiang University School of Medicine, Hangzhou, China; ^5^ Central Laboratory of Yongchuan Hospital, Chongqing Medical University, Chongqing, China

**Keywords:** carbapenem-resistant Klebsiella pneumoniae, antibiotic susceptibility, molecular epidemiology, whole-genome sequencing, ST1887, ceftazidime-avibactam

## Abstract

**Background:**

This study aimed to determine the molecular characteristics of carbapenem-resistant *Klebsiella pneumoniae* (CRKP) isolates in a hospital in western Chongqing, southwestern China.

**Methods:**

A total of 127 unique CRKP isolates were collected from the Yongchuan Hospital of Chongqing Medical University, identified using a VITEK-2 compact system, and subjected to microbroth dilution to determine the minimal inhibitory concentration. Enterobacteriaceae intergenic repeat consensus polymerase chain reaction and multilocus sequence typing were used to analyze the homology among the isolates. Genetic information, including resistance and virulence genes, was assessed using polymerase chain reaction. The genomic features of the CRKP carrying gene *bla*_KPC-2_ were detected using whole-genome sequencing.

**Results:**

ST11 was the dominant sequence type in the homology comparison. The resistance rate to ceftazidime-avibactam in children was much higher than that in adults as was the detection rate of the resistance gene *bla*_NDM_ (p < 0.0001). Virulence genes such as *mrkD* (97.6%), *uge* (96.9%), *kpn* (96.9%), and *fim-H* (84.3%) had high detection rates. IncF (57.5%) was the major replicon plasmid detected, and sequencing showed that the CRKP063 genome contained two plasmids. The plasmid carrying *bla*_KPC-2_, which mediates carbapenem resistance, was located on the 359,625 base pair plasmid IncFII, together with virulence factors, plasmid replication protein (*rep B*), stabilizing protein (*par A*), and type IV secretion system (T4SS) proteins that mediate plasmid conjugation transfer.

**Conclusion:**

Our study aids in understanding the prevalence of CRKP in this hospital and the significant differences between children and adults, thus providing new ideas for clinical empirical use of antibiotics.

## Introduction

Carbapenem-resistant *Klebsiella pneumoniae* (CRKP) has received increasing attention worldwide because of the widespread misuse of carbapenem antibiotics, with CRKP difficult to treat (Gong et al., 2018; Yungyuen et al., 2021). Several factors contribute to the development of CRKP resistance, among which carbapenemase-producing enzymes are the most predominant resistance mechanism (Conte et al., 2016; El-Badawy et al., 2020; Zhang et al., 2021). In 1996, the *bla*_KPC_ gene was first identified in the United States (Yigit et al., 2001). The CRKP producing KPC enzyme was the most common CRKP isolate in the global outbreak, causing serious endemic epidemics in Europe, America, Asia, and the Middle East (Munoz-Price et al., 2013; Aires-De-Sousa et al., 2019; Rodrigues et al., 2019; Yu et al., 2019). In China, the detection rate of *bla*_KPC-2_ is approximately 73% (Zhang et al., 2017). Previous studies have shown that the highest detection rate of *bla*_NDM_ among *K. pneumoniae* isolates was in China, where the detection rate was as high as 44.1% (Safavi et al., 2020). The first detection of the OXA-48 enzyme in CRKP was found in a urine sample from a Turkish patient (Poirel et al., 2004), and more than 50 countries reported the OXA-48 outbreak, including Taiwan, Zhejiang, and other areas in China (Lu et al., 2018; Shu et al., 2019).

A case of liver abscess caused by hypervirulent *K. pneumoniae* (hvKP) infection was first reported in 1986 in Taiwan Province, China (Liu et al., 1986). Since then, hvKP has been reported in many countries, including China, South Korea, Japan, Spain, Madagascar, Cambodia, and Senegal (Cubero et al., 2016; Zhang Y. et al., 2016; Harada et al., 2019; Yoon et al., 2019; Huynh et al., 2020; Parrott et al., 2021). A defining feature of hvKP is its hypermucoid appearance on agar plates. With its unique phenotypic (such as high mucus type and special serotype) and genotypic (such as carrying special virulence genes) characteristics, hvKP has a strong clinical pathogenicity. It can cause infection in patients with low immune function and severe community-acquired infection in young people with normal immune function, and can present migratory spread (Russo and Marr, 2019). HvKP has emerged because of several mechanisms, including pili, lipopolysaccharides, capsular polysaccharides, virulence factors and iron ingestion (Cheng et al., 2010; Lee et al., 2017; Chen et al., 2020). Patients infected with hvKP often show rapid symptom onset and an insidious disease course, making treatment more difficult (Shon and Russo, 2012; Zhang R. et al., 2016; Rahim et al., 2019). Resistance due to CRKP carrying virulence factors increases the failure rate of patient treatment. Therefore, understanding the virulence of CRKP in hospital isolates is vital.

Ceftazidime-avibactam (CAZ-AVI) is currently an effective treatment for carbapenem-resistant Enterobacteriaceae infection and has been approved for treating adults with complicated urinary tract infections (including pyelonephritis), complicated intra-abdominal infections, hospital-acquired pneumonia (including ventilator-associated pneumonia), and other infections caused by aerobic gram-negative bacteria. Avibactam (AVI), an enzyme inhibitor of triethylenediamine, does not contain a beta-lactam ring, and its enzyme inhibition spectrum is broad. AVI acylates the serine residue of beta-lactamase *via* noncovalent bonding with the beta-lactamase binding region, thus forming an active covalent compound, and the generated product does not undergo hydrolysis. Thus, the intervention has a long-acting enzyme inhibitory effect (Zhanel et al., 2013). In the CAZ-AVI combination, AVI protects the CAZ from degradation *via* various serine beta-lactamases, thus promoting CAZ-AVI activity. However, many cases of CAZ-AVI resistance have been reported since CAZ-AVI was approved in 2015 (Shields et al., 2016; Wang et al., 2020). The isolates producing metallo-β-lactamases such as *bla*_NDM_ are not active against CAZ-AVI (Zhang et al., 2020), and the detection rates of *bla*_NDM_ are different in different populations. Therefore, detecting the *in vitro* sensitivity of CAZ-AVI to CRKP would aid in understanding the clinical application of CAZ-AVI in CRKP treatment and predicting the application prospects of CAZ-AVI in future clinical treatments.

Sequence type 258 (ST258), an international high-risk epidemic CRKP lineage, is frequent in European and American countries (Wyres et al., 2020), whereas Sequence type 11 is the most common CRKP clone in China, and is part of the clonal complex 258 (CC258) and is a single-locus variant of ST258 (Liao et al., 2020).

We aimed to study a CRKP collection isolated at the Yongchuan Hospital of Chongqing Medical University, revealing their clinical characteristics, determining their resistance and virulence factors, and investigating the possible underlying resistance mechanisms.

## Materials and Methods

### Sample Collection

A total of 127 non-duplicate clinical CRKP isolates were collected between July 2018 and May 2020 from the Yongchuan Hospital at Chongqing Medical University, a major comprehensive medical center in western Chongqing province with 1480 beds in 47 wards. Age, sex, length of hospital stay, diagnosis, and patient outcomes were collected from the electronic medical records. All isolates were obtained from different clinical departments and were identified using a VITEK-2 automatic microbiological analyzer (bioMérieux, France) and preserved at -80°C for subsequent study. According to the Clinical and Laboratory Standards Institute guidelines (CLSI, 2020), the clinical isolates used in the present study were not susceptible to carbapenems (meropenem, imipenem, or ertapenem). The Ethics Committee determined that patient consent was not required because the present study was retrospective, and the identities of the patients were anonymized.

### Antibiotic Susceptibility Testing

Antibiotic susceptibility testing of each isolate was performed using a VITEK-2 compact automatic microbiological analyzer AST-GN card (bioMérieux). The minimum inhibitory concentration refers to the minimum concentration of the compound (µg/mL) required to stop bacterial growth as determined by the microbroth dilution method. Meropenem, imipenem, amikacin, levofloxacin, polymyxin B, tigecycline (TGC), and CAZ-AVI were used to determine the minimum inhibitory concentrations. ATCC 25922, ATCC 700603, and BAA-1705 were used as quality control isolates. The TGC results were interpreted based on the recommendations of the European Committee on Antimicrobial Susceptibility Testing (Testing, 2021), whereas the results for the other antibiotics were interpreted based on the CLSI (2020) criteria.

### Enterobacterial Repetitive Intergenic Consensus-Polymerase Chain Reaction (ERIC-PCR)

ERIC-PCR was used for homology analysis with the primer sequences ERIC-1: 5′- ATGTAAGCTCCTGGGGATTCAC-3′ and ERIC-2:5′- AAGTAAGTGACTGGGGTGAGCG-3′. The reaction conditions and system were as previously reported (Ranjbar and Mirsaeed Ghazi, 2013), and the amplified products were electrophoresed on a 1.5% agarose gel and photographed with a UV gel imaging system. The ERIC-PCR electrophoresis cluster analysis was performed using the NTSYS (V2.10e) software. The isolates with a > 90% similarity coefficient of the cluster dendrogram and no significant band differences were determined to have the same genotype.

### Multilocus Sequence Typing (MLST)

MLST was performed using seven housekeeping genes of *K. pneumoniae* that were amplified using primers from online databases (http://bigsdb.pasteur.fr/klebsiella/primers_used.html). PCR products were sequenced, and sequence types (STs) were determined using online database tools https://bigsdb.pasteur.fr/klebsiella/klebsiella.html.

### Resistance and Virulence Genes Molecular Detection

All resistance and virulence genes were detected using PCR, and the DNA was extracted using the boiling method (Srisrattakarn et al., 2017; Gong et al., 2018). We detected resistance genes, including carbapenemase genes (*bla*_KPC_, *bla*_NDM_, *bla*_IMP-4_, *bla*_IMP-8_, *bla*_VIM-1_, *bla*_VIM-2_, and *bla*_OXA-48_), AmpC beta-lactamase enzymes (*bla*_DHA_ and *bla*_ACC_), ESBL genes (*bla*_SHV_, *bla*_TEM_, *bla*_CTX-M-1_, and *bla*_CTX-M-9_), and quinolone resistance genes (*qnrA*, *qnrB*, *qnrS*, *qepA*, and *aac(6’)Ib-cr*). The virulence genes detected included *fim-H*, *magA*, *aero*, *alls*, *iroNB*, *kpn*, *mrkD*, *rmpA*, *uge*, and *wcaG*. All primers were obtained from previous studies (Gay et al., 2006; Park et al., 2006; Ma et al., 2009; El Fertas-Aissani et al., 2013; Compain et al., 2014; Wasfi et al., 2016; Jian-Li et al., 2017; Fu et al., 2018; Gong et al., 2018). Positive amplification products were sequenced, and the sequencing results were compared using the Basic Local Alignment Search Tool (BLAST) at https://blast.ncbi.nlm.nih.gov/Blast.cgi.

### Identification of Plasmids by PCR Based Replicon Typing (PBRT)

PBRT was performed to classify the plasmids into various incompatibility groups according to a previously described method (Carattoli et al., 2005). Replicons from 18 major plasmid families in Enterobacteriaceae were searched using PBRT: IncK, IncX, IncY, IncF, IncT, IncP, IncI1, IncFIIA, IncFIB, IncL/M, IncN, IncHI2, IncFIA, IncA/C, IncW, IncHI1, IncFIC, and IncB/O. Positive amplification products were sequenced, and the sequencing results were compared using BLAST.

### Whole-Genome Sequencing and Analysis

Bacterial genomic DNA was extracted using a MagAttract HWM DNA Mini Kit (cat. no. 67563) from Qiagen. The samples were subjected to third-generation (Nanopore) and second-generation (Illumina Hiseq) whole-genome sequencing by Shanghai Yuanxin Biological Medicine Technology Co., Ltd. Gene prediction for the bacteria was performed using Glimmer3.02. The resistance and virulence genes were identified after the assembled genome was uploaded to ResFinder and the Virulence Factor Database. The PlasmidFinder Database and BLASTn were used to identify the incompatibility groups. Finally, chromosomal and plasmid maps were drawn using the CGView online tool and BRIG (v0.95) respectively. The genetic structure surrounding *bla*_KPC-2_ was drawn using Easyfig (v2.2.5). The CRKP063 genomic sequence has been deposited in GenBank under accession no. MZ156798.

### Statistical Analysis

Statistical differences among groups were determined using Chi-square and Fisher’s exact tests in SPSS 23.0, and statistical significance was set at p < 0.05.

## Results

### Analysis of Clinical Information

A total of 127 unique isolates were obtained from 21 hospital departments; 13 isolates were from children and the remainder were from adults. Among these isolates, 56 (44.1%) were from the intensive care unit (ICU), 13 (10.2%) were from the respiratory medicine department, 10 (7.9%) were from the respiratory and critical care medicine department, nine (7.1%) were from the neonatal unit, and the remainder were from the rehabilitation medicine, hepatobiliary surgery, geriatrics, rheumatology, kidney disease, and endocrine departments. Two (1.6%) isolates came from the pediatric ICU, neurological ICU, hematology, infectious disease department, pediatric, orthopedic, and urology departments, and one isolate was obtained from the neurology, neurosurgery, cardiovascular, gastrointestinal surgery, and medical oncology departments. Regarding sources, 72 (56.7%) isolates were obtained from sputum, 23 (18.1%) were obtained from urine, and 19 (15.0%) were obtained from bronchoalveolar lavage fluid; the remainder were obtained from blood, secretions, puncture fluid, and superficial surgery. Most patients had pulmonary disease and were treated with invasive procedures, such as endotracheal intubation or invasive ventilation. The mortality and improvement rates of patients after infection were 8.7% (11/127) and 33.9% (43/127), respectively. Based on the clinical features summarized. most patients with CRKP (61.4%) had used carbapenems.

### Results of Antibiotic Susceptibility Testing

**Figure 1:**
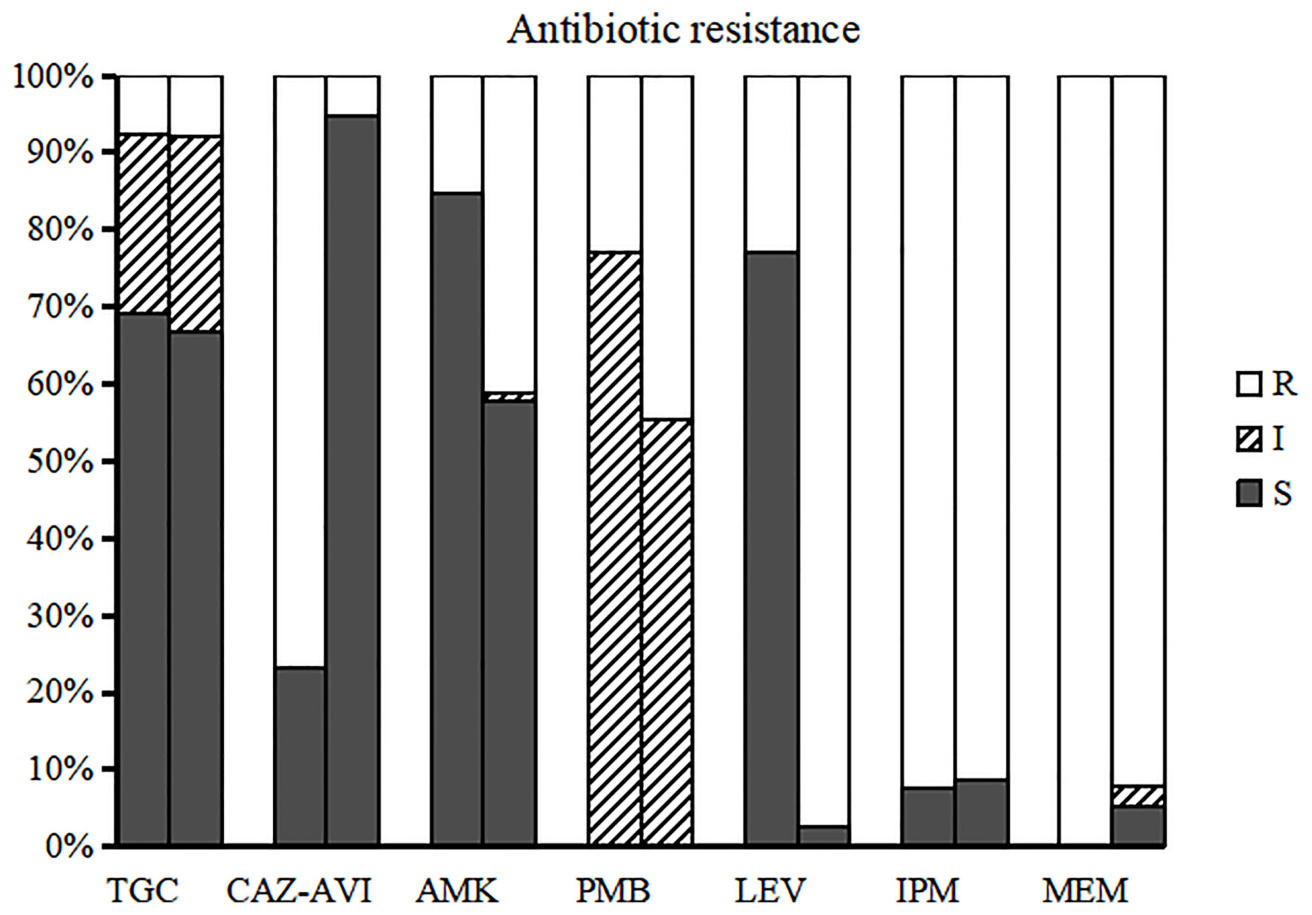
Susceptibility of CRKP to different antimicrobial agents in children and adults. The left bar for each group represents the antibiotic resistance of children and the right bar represents adults. TGC, tigecycline; CAZ-AVI, ceftazidime-avibactam; AMK, amikacin; PMB, polymyxin B; LEV, levofloxacin; IPM, imipenem; MEM, meropenem.

Based on the susceptibility testing results, all isolates were identified as multidrug-resistant, defined as resistant to three or more antibiotic classes (Magiorakos et al., 2012), In addition to the high resistance rate of carbapenem antibiotics, CRKPs had high resistance rate to quinolones. The antibiotic resistance rates of polymyxin B, amikacin, and TGC were 42.5%, 38.6%, and 7.9%, respectively. A total of 16 isolates (12.6%) showed resistance to CAZ-AVI, 10 from children (76.9%) and six from adults (5.6%) (p < 0.0001). The susceptibility of CRKP to different antibiotics between children and adults indicated that the resistance rate of CRKP to levofloxacin in adults was significantly higher than that in children (p < 0.05) (**Figure 1**).

### Homology Comparison

**Figure 2:**
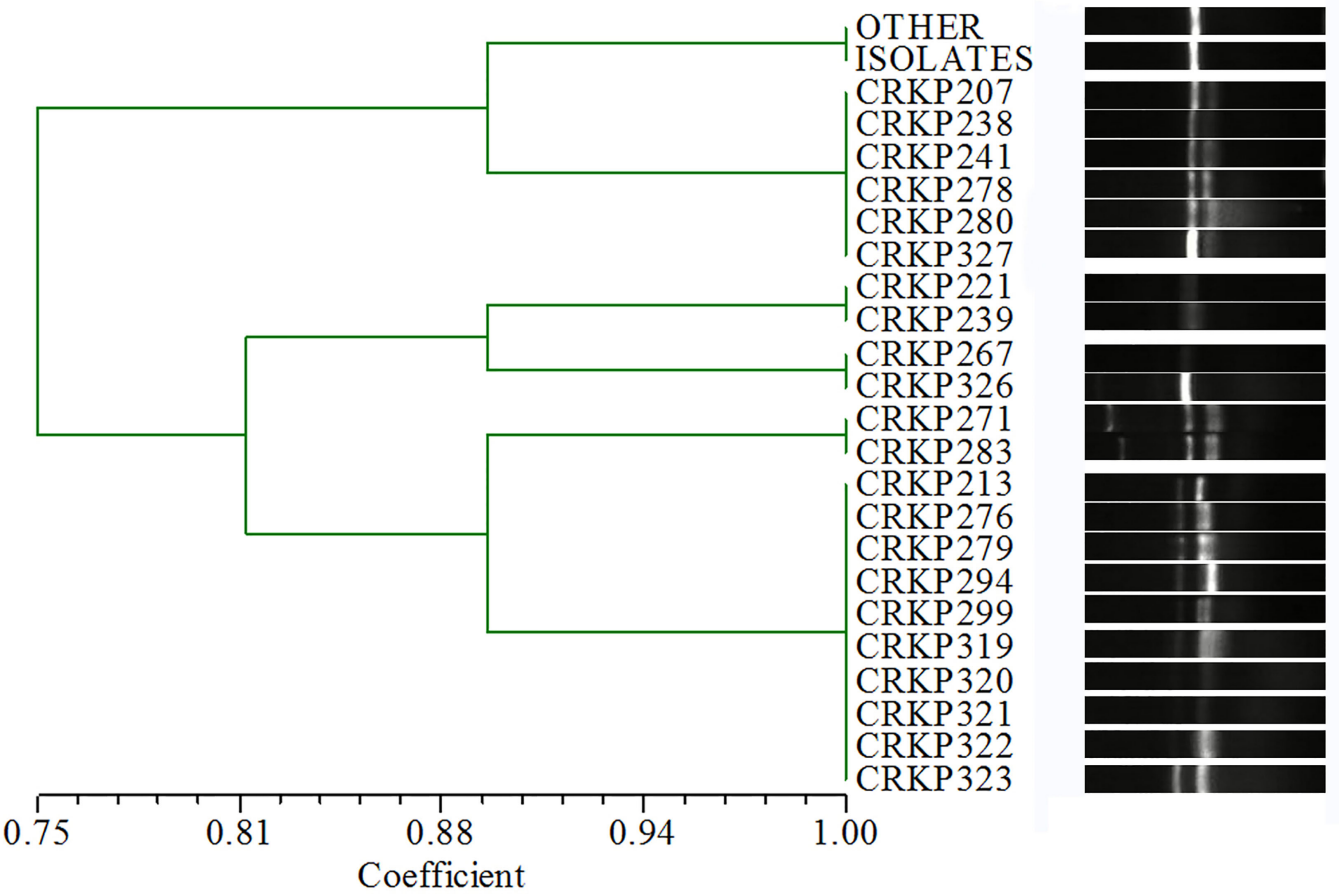
ERIC-PCR results. The isolates with > 90%similarity coefficient for the cluster dendrogram and no significant band difference were determined to have the same genotype. “OTHER ISOLATES” refers to 105 isolates in addition to the 22 isolates listed in the figure.

A total of 19 STs were identified among the existing STs in all isolates: 103 (81.1%) belonged to ST11, four (3.1%) belonged to ST1887, three (2.4%) belonged to ST617, two (1.6%) belonged to ST664, and one belonged to each of the other STs. Based on the ERIC-PCR results (**Figure 2**, **Supplementary Figures**), six different genotypes (A–F) were observed: 105 (82.7%) were type A, 10 (7.9%) were type B, six (4.7%) were type C, and three pairs were type D, E, and F. The MLST and ERIC-PCR results were not completely consistent; however, both detection methods showed high homology of CRKP in the study hospital.

### Detection of Resistance and Virulence Genes

**Figure 3:**
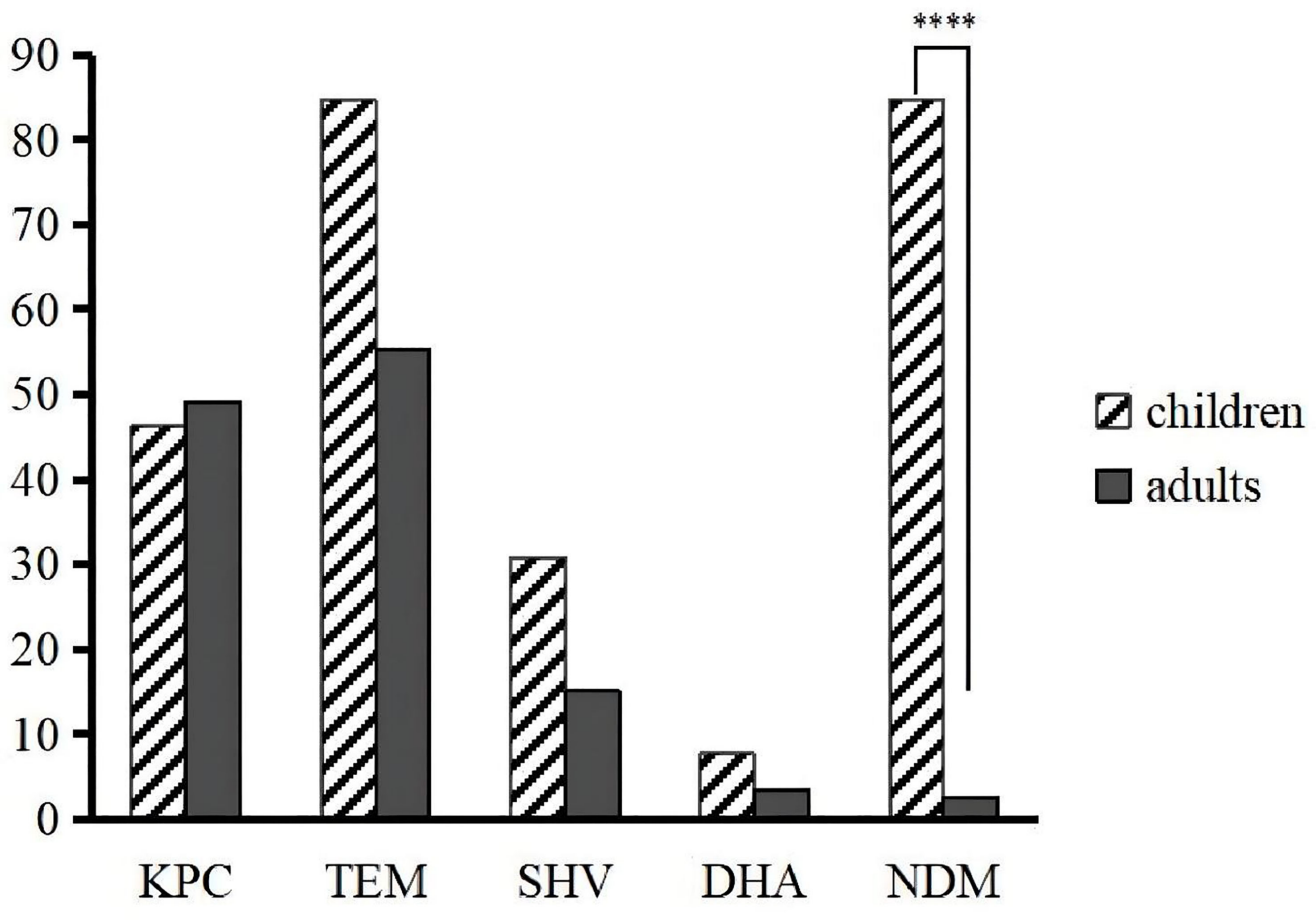
Differences in the prevalence of resistance genes between children and adults. ****p < 0.0001.

Carbapenem resistance genes such as *bla*_KPC-2_ (87.4%), *bla*_NDM_ (11.0%), and *bla*_IMP-4_ were detected. *bla*_TEM_ (79.5%) and *bla*_SHV_ (56.7%) also had high detection rates. Other ESBLs, such as *bla*_CTX-M-1_ (2.4%), *bla*_CTX-M-9_ (25.2%) and *bla*_CTX-M-65_ (24.4%), which is a common allelic variant of *bla*_CTX-M-9_, were detected. *bla*_DHA_ (3.9%), an AmpC beta-lactamase, was also detected. *qnrB* (2.4%), *qnrS* (13.4%), and *aac(6’)-Ib-cr* (7.1%) were detected among the plasmid-mediated quinolone resistance genes. Most differences in the detection rates of metalloenzymes or serinases, including *bla*_KPC-2_, were not statistically significant between children and adults, except for *bla*_NDM_, whose detection rate in children was significantly higher than that in adults (p < 0.0001) (**Figure 3**). Every isolate in our study had at least two resistance genes detected, except for three isolates with only *bla*_TEM_.

Regarding virulence genes, *mrkD*, *uge*, *kpn*, and *fim-H* had high detection rates in each ST classification. *rmpA* and *allS* were detected, including 100% ST1887 isolates with *allS*. Both *aero* (1.6%) and *wcaG* (1.6%) were detected twice. *iroNB* and *magA* were not detected in the present study. All isolates were detected to have at least three virulence genes, and the co-detection rate of *fim-H*, *kpn*, *mrkD*, and *uge* was as high as 63.0%. *rmpA* and *aero* were detected simultaneously in two isolates (**Table 1**).

**Table 1:** Resistance and virulence genes in different STs.

		ST11(*n*=103)	ST1887(*n*=4)	ST617(*n*=3)	ST664(*n*=2)	Other STs (*n*=15)
**Resistance Genes**						
	**AmblerA**					
	* KPC-2*	98 (95.1%)	0 (0%)	1 (33.3%)	2 (100%)	10 (66.7%)
	* SHV*	65 (63.1%)	2 (50%)	0 (0%)	1 (50%)	4 (26.7%)
	* TEM*	79 (76.7%)	4 (100%)	3 (100%)	0 (0%)	15 (100%)
	* CTX-M-1*	2 (2.0%)	0 (0%)	0 (0%)	0 (0%)	1 (1.0%)
	* CTX-M-9*	32 (31.1%)	0 (0%)	0 (0%)	0 (0%)	1 (1.0%)
	**AmblerB**					
	* NDM*	0 (0%)	4 (100%)	3 (100%)	0 (0%)	7 (46.7%)
	* IMP-4*	0 (0%)	0 (0%)	0 (0%)	0 (0%)	1 (1.0%)
	* IMP-8*	0 (0%)	0 (0%)	0 (0%)	0 (0%)	0 (0%)
	* VIM-1*	0 (0%)	0 (0%)	0 (0%)	0 (0%)	0 (0%)
	* VIM-2*	0 (0%)	0 (0%)	0 (0%)	0 (0%)	0 (0%)
	**AmblerC**					
	* DHA*	4 (3.9%)	0 (0%)	0 (0%)	1 (1.0%)	0 (0%)
	* ACC*	0 (0%)	0 (0%)	0 (0%)	0 (0%)	0 (0%)
	**AmblerD**					
	* OXA-48*	0 (0%)	0 (0%)	0 (0%)	0 (0%)	0 (0%)
**Virulence Genes**						
	*uge*	99 (96.1%)	4 (100%)	3 (100%)	2 (100%)	15 (100%)
	*rmpA*	12 (11.7%)	0 (0%)	0 (0%)	0 (0%)	3 (20%)
	*magA*	0 (0%)	0 (0%)	0 (0%)	0 (0%)	0 (0%)
	*fimH*	83 (80.6%)	4 (100%)	3 (100%)	2 (100%)	15 (100%)
	*kpn*	100 (97.1%)	3 (75%)	3 (100%)	2 (100%)	15 (100%)
	*mrkD*	100 (97.1%)	4 (100%)	3 (100%)	2 (100%)	15 (100%)
	*aero*	0 (0%)	0 (0%)	0 (0%)	0 (0%)	2 (13.3%)
	*wcaG*	0 (0%)	0 (0%)	0 (0%)	0 (0%)	2 (13.3%)
	*allS*	0 (0%)	4 (100%)	0 (0%)	0 (0%)	2 (13.3%)
	*iroNB*	0 (0%)	0 (0%)	0 (0%)	0 (0%)	0 (0%)

### Identification of Plasmid Incompatibility Groups Using PBRT

PBRT method revealed that 84 (66.1%) plasmid DNAs belonged to single replicon plasmids, including IncF in 73 (57.5%) isolates, IncFIIA in 10 (7.9%) isolates, and IncN in one isolate. A total of 19 (15.0%) isolates carried multiple Inc groups of plasmids, and IncF + IncFIIA was the only combination detected. No Inc groups could be detected in 24 (18.9%) of isolates. Among the 99 ST11 isolates carrying *bla*_KPC-2_, 66 (66.7%) carried IncF plasmids (**Table 2**).

**Table 2:** Incompatible plasmids in different ST isolates carrying *bla*_KPC-2_ or *bla*_NDM_.

		IncF	IncFIIA	IncF+IncFIIA	IncN	None
Isolates carrying *bla*_KPC-2_						
	ST11 (*n*=99)	66 (66.7%)	2 (2.0%)	18 (18.2%)	0 (0%)	13 (13.1%)
	ST664 (*n*=2)	0 (0%)	0 (0%)	0 (0%)	0 (0%)	2 (100%)
	ST617 (*n*=1)	0 (0%)	0 (0%)	0 (0%)	0 (0%)	1 (100%)
	ST1887 (*n*=0)	0 (0%)	0 (0%)	0 (0%)	0 (0%)	0 (0%)
	Other STs (*n*=12)	3 (25.0%)	2 (16.7%)	1 (8.3%)	0 (0%)	6 (50.0%)
Isolates carrying *bla*_NDM_						
	ST11 (*n*=0)	0 (0%)	0 (0%)	0 (0%)	0 (0%)	0 (0%)
	ST1887 (*n*=4)	0 (0%)	3 (75.0%)	0 (0%)	0 (0%)	1 (25.0%)
	ST617 (*n*=3)	0 (0%)	0 (0%)	0 (0%)	0 (0%)	3 (100%)
	ST664 (*n*=0)	0 (0%)	0 (0%)	0 (0%)	0 (0%)	0 (0%)
	Other STs (*n*=7)	1 (14.3%)	3 (42.9%)	0 (0%)	0 (0%)	3 (42.9%)

### Genome Sequencing and Analysis

**Figure 4:**
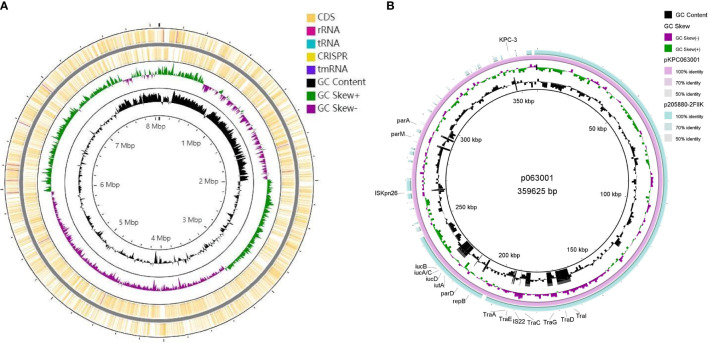
Schematic circular genome of CRKP063. **(A)** Chromosome circle map. From the outside to the inside, the first two circles represent the coding sequences on the positive and negative chains. The third circle represents the GC skew value. When the value was positive, the positive chain was more likely to transcribe the CDS, and when it was negative, the negative chain was more likely to transcribe the CDS. The fourth circle represents the GC content, and the outer part indicates that the GC content in this region was higher than the average GC content of the whole-genome. **(B)** Circle map of the IncFII plasmid carrying the *bla*_KPC-2_ gene. The outermost circle represents the plasmid p205880-2FIIK (accession no. MN824002.1), possessing high similarity.

**Figure 5:**
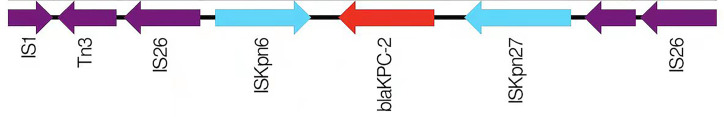
A schematic diagram of the genetic structure surrounding *bla*_KPC-2_. The direction of the arrow represents the direction of transcription.

CRKP063, an isolate carrying *bla*_KPC-2_, was subjected to genome sequencing and analysis. After nanopore sequencing and filtering of the raw data, the bacterium had 25,901,842 reads with 9,208,426 base pairs (bp), a G + C content of 60.4%, and 7701 annotated protein-coding sequences. The CRKP063 genome consisted of a circular chromosome of 8,074,931 bp and two plasmids. Resistance genes to aminoglycosides (*aph(3´)-IIb*), fosfomycin (*fosA*), phenicols (*catB7*), and beta-lactams (*bla*_KPC-2_, *bla*_OXA-396_ and *bla*_SHV-182_) were identified using whole-genome sequencing. According to our analysis, 37 rRNA genes, 110 tRNA genes, and 42,108 putative open reading frame genes were present on the circular chromosome (**Figure 4A**). CRKP063 contained a 359,625 bp IncFII plasmid with the siderophore aerobactin genes (*iutA* and *iucABCD*); plasmid replication protein (*rep B*); stabilizing protein (*par A*); and type IV secretion system (T4SS) proteins *traA*, *traB*, *traC*, *traD*, and *traM*, which mediate plasmid conjugation transfer. BLAST comparison revealed similarity of 71% between the plasmid and p205880-2FIIK (accession no. MN824002.1) (**Figure 4B**). Comparison of the regions surrounding *bla*_KPC-2_ revealed that the plasmid contained multiple mobile genetic elements, including IS*kpn27*, IS*kpn6*, Tn*3*-like transposons, and two copies of IS*26* (**Figure 5**). Outside this plasmid, CRKP063 contained other virulence-associated genes, including adhesin gene type 1 fimbriae (*fim-H*) and type 3 fimbriae (*mrkACDF*).

## Discussion

As expected, the department with the highest prevalence of CRKP was the ICU, and the major source was sputum (Zhang et al., 2021). The duration of hospital stay in the same department might indicate that the isolates were disseminated within the departments. All mortality occurred in the ICU (10/11) or respiratory and critical care medicine departments; however, the patients rarely had underlying diseases. We hypothesized that mortality was closely associated with complications.

Among the 13 children and 114 adults, the resistance rate to CAZ-AVI was significantly higher in children than in adults. Therefore, in the clinical use of CAZ-AVI, attention must be paid to this result. In addition, the detection rate of *bla*_NDM_ in children was significantly higher than that in adults. Our results are similar to those of a previous study indicating that acquiring a *bla*_NDM-5_-harboring plasmid led to resistance to CAZ-AVI in KPC-2-producing *K. pneumoniae* during treatment (Huang et al., 2021). With the CAZ-AVI combination, AVI protects CAZ from degradation by various serine beta-lactamases, thus promoting CAZ-AVI activity. Therefore, the two isolates that contained only *bla*_KPC_ and *bla*_TEM_ but were resistant to CAZ-AVI require further investigation.

Polymyxins (including colistin and polymyxin B) are the last resort for treating carbapenem-resistant Enterobacteriaceae infections (Macesic et al., 2020); therefore, the resistance in 42.5% of isolates in our study was concerning. A plasmid-mediated polymyxin resistance gene, *mcr-1*, was first reported in 2015 (Liu et al., 2016), and China had the highest prevalence of *mcr-*positive isolates (Nang et al., 2019). However, *mcr-1* was not detected in any of the isolates. The reasons for this resistance require further study.

Regarding resistance genes, most (98.4%) isolates had more than two genes, indicating that the multiple resistance of CRKP may be because of multiple gene interactions. In the present study, *bla*_KPC-2_ was the main carbapenem resistance gene, which agrees with the current prevalence (Hu et al., 2020). Furthermore, the environment surrounding *bla*_KPC-2_ as IS*26*-*tnpR*-IS*kpn27*-*KPC-2*-IS*kpn6*-IS*26*-Tn*3* was different from the transposon Tn*4401* popular abroad (Bowers et al., 2015) and the transposon Tn*1721* popular in china (Wang et al., 2015; Yang et al., 2020). It is a typical plasmid-mediated antibiotic resistance gene that is widely distributed in plasmids of different sizes and types (Jain et al., 2013; Pitout et al., 2015), including IncF, IncI, IncA/C, IncN, IncX, IncR, IncP, IncU, IncW, IncL/M, and ColE (Chen et al., 2014). Based on the differences in plasmid replication, *bla*_KPC_ exists mainly in the IncF plasmid in eastern China (Hu et al., 2020). Our results showed that when *bla*_KPC-2_ and plasmids were detected simultaneously, the plasmid was IncF; therefore, the results were consistent with those from previous studies. IncF + IncFIIA appeared in all isolates carrying *bla*_KPC-2_, indicating that plasmids have strong mobility and can be transferred between isolates, causing that spread and diffusion of antibiotic resistance.

KPC resistance plasmids simultaneously contain various virulence and retention factors, thus significantly improving the adaptability of KPC-producing strains to the external environment and facilitating the spread of KPC (Dong et al., 2018). *uge*, *mrkD*, *kpn*, and *fim-H* were present in almost all CRKP isolates in the present study. A previous study showed that *fim-H* and *mrkD* encode distinct types of fimbriae that often cause respiratory and urinary tract infections (Yan et al., 2016). Our clinical data showed that 79.0% of the patients had lung diseases, and 71.7% of isolates were from the respiratory tract. Several studies have indicated that isolates coexpressing the mucus phenotype coding gene (*rmpA*) and aerobin gene (*aero*) can be considered hvKP (Siu et al., 2012). Two isolates in our study, belonging to ST1373 and ST23 can be defined as hvKP based on this condition. The detection rate of hvKP in the present study was only 1.6%, indicating that the CRKP isolates in this hospital were not yet highly virulent. This result is consistent with those of our previous study in the ICU (Zeng et al., 2021). Several studies have observed that hvKP over time has been susceptible to antibiotics and related mainly to community-acquired infections (Klaper et al., 2021). However, there are reports where this has been reversed and hvKP with antibiotic resistance and involved in infections associated with hospital care are gradually beginning to appear (Gu et al., 2018).

Based on a comparison of MLST and ERIC-PCR, we concluded that all isolates from the hospital had a high degree of homology, particularly in ST11 (81.1%), in agreement with the prevalence in China (Qi et al., 2011; Liao et al., 2020). ST11 has recently been reported elsewhere in China, causing fatal infections and high mortality rates in other hospitals (Hu et al., 2020; Qiao et al., 2020). We concluded that all isolates had high homology based on the similarity coefficients of different clusters by ERIC-PCR. One novel allele of locus *infB* in MLST was found in the present study, which was defined as *infB*-236. In addition, three new STs were identified. ST1887 CRKP is a ST commonly found in our hospital, accounting for 3.1% of cases. All four ST1887 isolates expressed the *bla*_NDM_ and *bla*_TEM_ resistance genes, and the virulence genes of *uge*, *fim-H*, *mrkD*, and *allS*, which were higher than those in ST11 CRKP, particularly *bla*_NDM_ (p < 0.0001) and *allS* (p < 0.0001) (**Table 1**).

In summary, ST11 CRKP isolates with *bla*_KPC-2_ remain widespread in western Chongqing, southwestern China, and transmission within the hospital should be monitored, and effectively controlled. Effective measures should be taken to prevent further expansion of multi-drug resistant bacteria.

## Data Availability Statement

The datasets presented in this study can be found in online repositories. The names of the repository/repositories and accession number(s) can be found below: https://www.ncbi.nlm.nih.gov/, MZ156798.

## Ethics Statement

The studies involving human participants were reviewed and approved by The Ethics Committee of Yongchuan Hospital of ChongQing Medical University. Written informed consent to participate in this study was provided by the participants’ legal guardian/next of kin.

## Author Contributions

BL and XZ conceived of and designed the study. WH and JZ wrote this paper and contributed equally to this work. LZ, CY, LY, and KH performed the experiments. JW, JL, and XL analyzed the data. All authors contributed to the article and approved the submitted version.

## Funding

This work was supported by General projects of Chongqing Natural Science Foundation (cstc2020jcyj-msxm0067), Yongchuan Natural Science Foundation (2021yc-jckx20053) and Talent introduction project of Yongchuan Hospital of Chongqing Medical University (YJYJ202005, YJYJ202004).

## Conflict of Interest

The authors declare that the research was conducted in the absence of any commercial or financial relationships that could be construed as a potential conflict of interest.

## Publisher’s Note

All claims expressed in this article are solely those of the authors and do not necessarily represent those of their affiliated organizations, or those of the publisher, the editors and the reviewers. Any product that may be evaluated in this article, or claim that may be made by its manufacturer, is not guaranteed or endorsed by the publisher.
