# Supplementary figures and images for: Carbapenemase Production and Epidemiological Characteristics of Carbapenem-Resistant Klebsiella pneumoniae in Western Chongqing, China
Source: Front Cell Infect Microbiol. 2022 Jan 4;11:775740. doi: 10.3389/fcimb.2021.775740 (PMC8769044; doi:10.3389/fcimb.2021.775740)

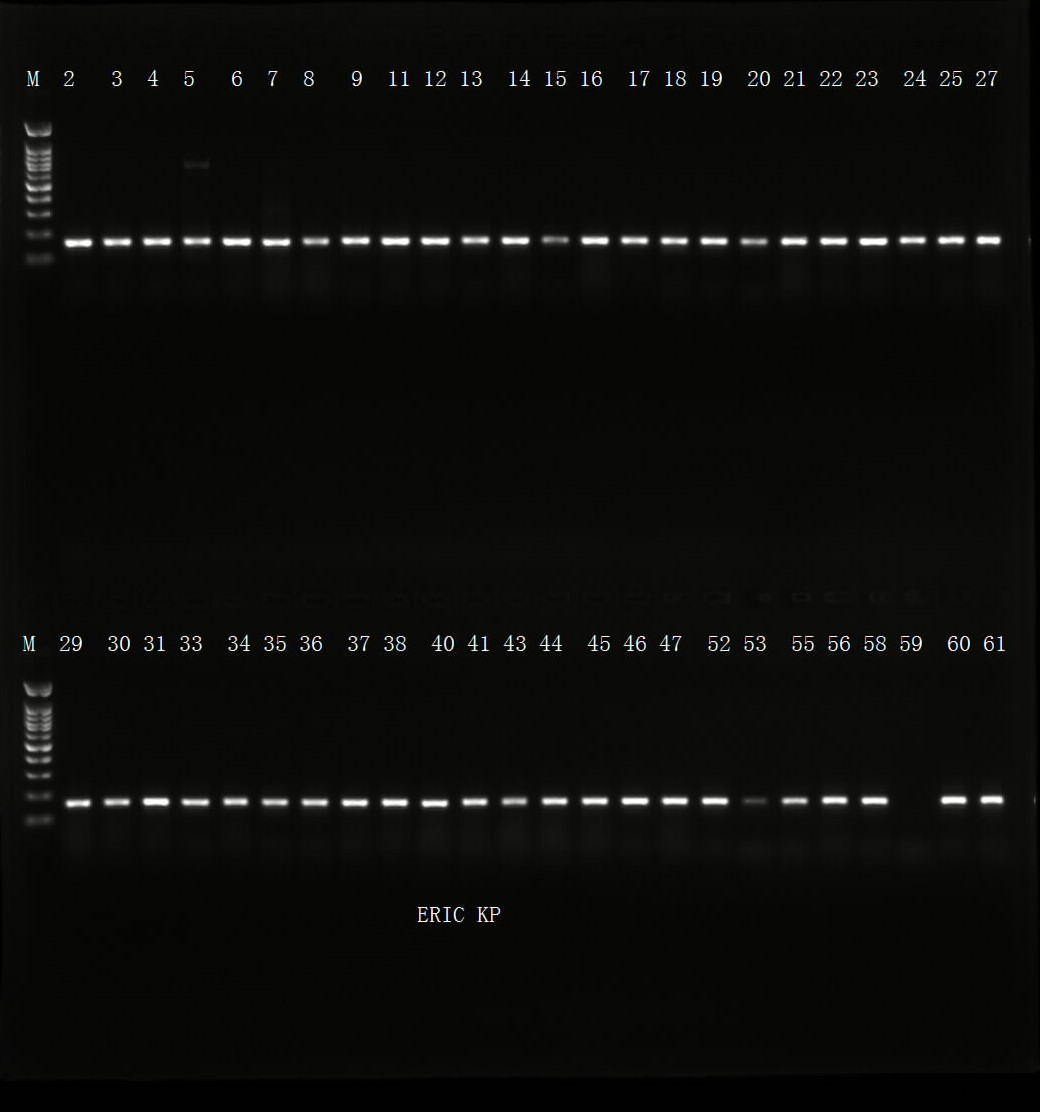

Supplement: Supplementary file 1 [file DataSheet_1.zip › Supplementary Figures-Electrophoresis results of ERIC-PCR/Isolates numbers CRKP002 to CRKP061..JPG]

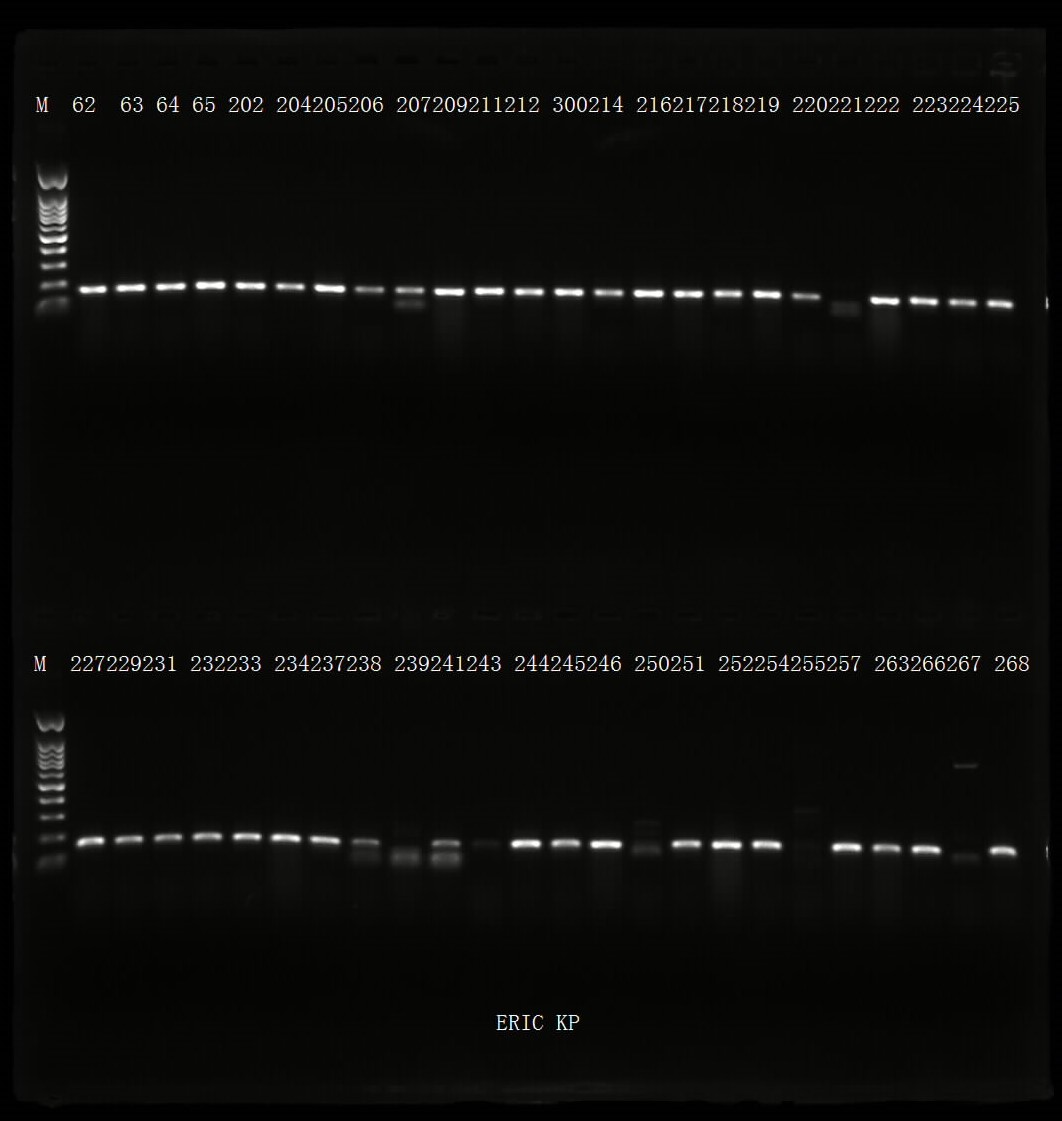

Supplement: Supplementary file 1 [file DataSheet_1.zip › Supplementary Figures-Electrophoresis results of ERIC-PCR/Isolates numbers CRKP062 to CRKP268..JPG]

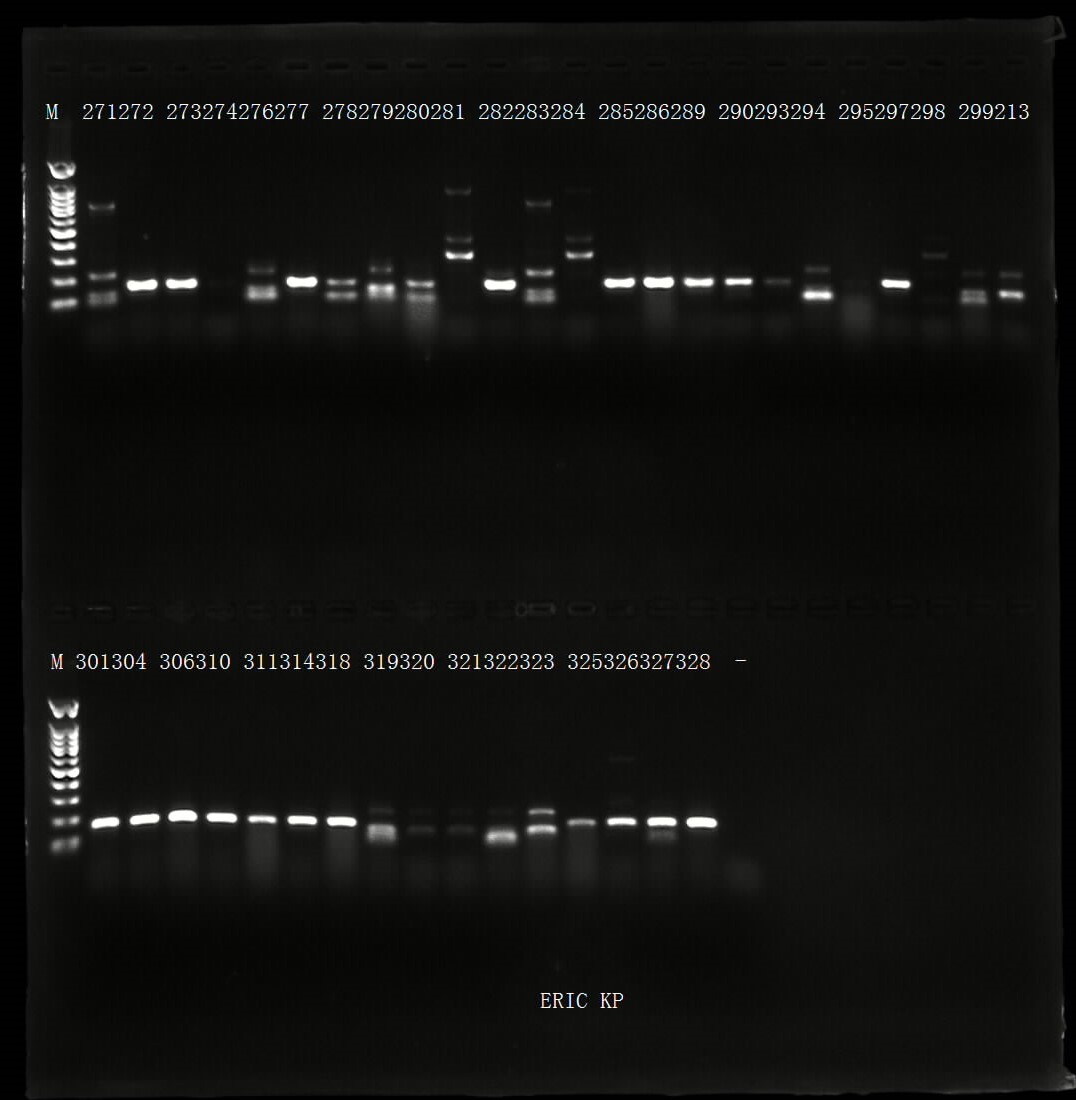

Supplement: Supplementary file 1 [file DataSheet_1.zip › Supplementary Figures-Electrophoresis results of ERIC-PCR/Isolates numbers CRKP271 to CRKP328..JPG]
